# Revisiting sex as a biological variable in hypertension research

**DOI:** 10.1172/JCI180078

**Published:** 2024-09-03

**Authors:** Michael J. Ryan, John S. Clemmer, Roy O. Mathew, Jessica L. Faulkner, Erin B. Taylor, Justine M. Abais-Battad, Fiona Hollis, Jennifer C. Sullivan

**Affiliations:** 1 Columbia VA Health Care System, Columbia, South Carolina, USA.; 2 University of South Carolina School of Medicine, Columbia, South Carolina, USA.; 3 University of Mississippi Medical Center, Jackson, Mississippi, USA.; 4 Loma Linda VA Health Care System, Loma Linda, California, USA.; 5 Medical College of Georgia at Augusta University, Augusta, Georgia, USA.

## Abstract

Half of adults in the United States have hypertension as defined by clinical practice guidelines. Interestingly, women are generally more likely to be aware of their hypertension and have their blood pressure controlled with treatment compared with men, yet hypertension-related mortality is greater in women. This may reflect the fact that the female sex remains underrepresented in clinical and basic science studies investigating the effectiveness of therapies and the mechanisms controlling blood pressure. This Review provides an overview of the impact of the way hypertension research has explored sex as a biological variable (SABV). Emphasis is placed on epidemiological studies, hypertension clinical trials, the genetics of hypertension, sex differences in immunology and gut microbiota in hypertension, and the effect of sex on the central control of blood pressure. The goal is to offer historical perspective on SABV in hypertension, highlight recent studies that include SABV, and identify key gaps in SABV inclusion and questions that remain in the field. Through continued awareness campaigns and engagement/education at the level of funding agencies, individual investigators, and in the editorial peer review system, investigation of SABV in the field of hypertension research will ultimately lead to improved clinical outcomes.

## Introduction

Hypertension increases all-cause mortality, and blood pressure (BP) is a major modifiable risk factor for the development of cardiovascular disease (CVD) and renal disease. Options for the treatment of hypertension were limited until the 1950s and 1960s, when diuretics, β-adrenergic blockers (beta blockers), and mineralocorticoid receptor antagonists were first approved (1–3). Angiotensin-converting enzyme (ACE) inhibitors and calcium channel blockers (CCBs) became available in the 1970s and 1980s, respectively, followed by angiotensin receptor blockers (ARBs) in the 1990s (1, 2). The renin inhibitor aliskiren was approved in 2007 (4); radiofrequency ablation of the renal nerves continues to be explored in clinical trials (5); and the first endothelin antagonist was recently approved for patients with resistant hypertension (6). The first-line treatment of hypertension remains an ACE inhibitor, ARB, CCB, or thiazide diuretic, an approach that is more than 50 years old. Despite the availability of antihypertensive therapies, a high percentage of hypertensive patients do not achieve BP control, even when taking multiple standard-of-care therapeutics (7).

Consideration of sex as a biological variable (SABV) in the design, analysis, and reporting of clinical and preclinical studies is critical, as there are clear differences in BP between male and female individuals. The Framingham Heart Study more than 35 years ago showed that the two-year incidence of hypertension was lower in women than men until women reached their 50s, at which time incidence of hypertension was higher in women (8). Nevertheless, the female sex remains underrepresented in clinical trials, including hypertension trials, where women account for 38% of enrolled participants (9). Underrepresentation of female individuals also occurs in preclinical studies. Beery and Zucker (10) showed that female participants are not sufficiently included in the design of preclinical studies across biological disciplines. Even after the NIH implemented its policy requiring consideration of SABV in experimental design in 2015, we remain far from the goal of equitable female inclusion (11). This oversight limits the rigor and reproducibility of scientific work and ultimately delays progress toward better health care for all.

At present, there are no sex-specific considerations for the treatment of hypertension, and current guidelines do not suggest differential thresholds for treatment between the sexes (12). This is potentially problematic, considering that there are sex-specific side effects of antihypertensive therapies, sex differences in therapeutic compliance, and a lack of guidelines to treat hypertension in female-specific (i.e., pregnancy, postmenopause) or female-biased (e.g., chronic autoimmune disorders) conditions. Moreover, women were underrepresented in clinical trials for the standard-of-care therapeutics (13). Studies that include real-world datasets suggest that BP is better controlled in men than women, while population research datasets, which often exclude older women, conclude that BP control is better in women (12).

The goal of this Review is to provide an overview of the impact of SABV on hypertension research and identify key questions remaining in the field, with an emphasis on epidemiological studies, hypertension clinical trials, the genetics of hypertension, sex differences in immunology and gut microbiota during hypertension, and the impact of sex on the central control of BP. The focus of this Review is on sex differences as defined by chromosomal complement, as opposed to examining the impact of gender, which is a societal construct.

## Epidemiology of hypertension and related diseases

Nearly half of adults in the US have hypertension, defined as a BP of at least 130 mmHg systolic blood pressure (SBP) or at least 80 mmHg diastolic blood pressure (DBP). High BP is an important modifiable risk factor for heart disease, accounting for 120,000 deaths in the US annually (14). Sex differences in the development and progression of hypertension are well documented, with women having a lower prevalence of hypertension at younger ages compared with men but a higher prevalence later in life. Specifically, the prevalence of hypertension in women aged 20–34 years is approximately half (15%) that of age-matched men (28%). Although National Health and Nutrition Examination Survey (NHANES) data change with each survey, it is clear that the prevalence of hypertension rapidly increases in women older than 60 years, ultimately matching or exceeding the prevalence in men (14). While the prevalence of hypertension is greater in men overall (50.4% vs. 43%, age ≥20 years), hypertension-related mortality overall is higher in women (51.3%) compared with men (48.7%) (14). Table 1 summarizes the prevalence and mortality of hypertension and hypertension-related diseases in men and women.

**Figure 1:**
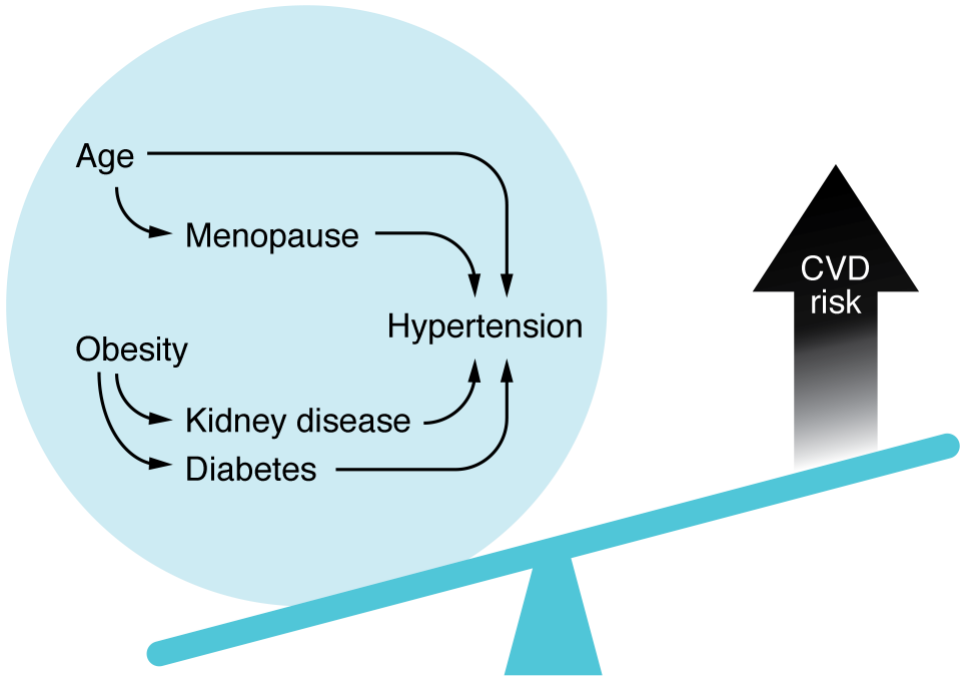
Increased CVD risk in women is driven by many factors, including age, menopause, and obesity.

Postmenopausal mechanisms, and comorbidities such as obesity, are commonly proposed to underly the increased prevalence of hypertension in postmenopausal women. Being overweight accounts for approximately 60% of hypertensive cases in women, compared with 34% in men (15). Racial and ethnic differences likely also exist within the two sexes. African American individuals tend to have a higher prevalence of obesity than White Americans, which may contribute to the greater hypertension burden among African Americans. The Coronary Artery Risk Development in Young Adults (CARDIA) study followed young normotensive individuals for up to 30 years to examine predictors of hypertension. While White women had a lower incidence of hypertension before age 55 (40%) than White men (55%), African American men and women had a higher incidence of hypertension (76%) (16). Others reported a similar overall prevalence when adjusted for age: White women had the lowest prevalence of hypertension (40%), followed by White men (47%), with African American men and women having the highest prevalence (~57%) (17). There is a continued need to characterize the interactions of sex, obesity, and race in hypertension and CVD (Figure 1).

### Chronic kidney disease.

Hypertension is a major risk factor for chronic kidney disease (CKD). While more women are diagnosed with CKD than men, men with CKD have worse outcomes (18), including a more rapid decline in renal function, greater proteinuria, and quicker progression to end-stage renal disease (19). Men with CKD also have a higher risk of cardiovascular and all-cause mortality than women with CKD (20). Women are less likely to be diagnosed with CKD than men, which could delay treatment and decrease quality of life (19). This is especially true for women with type 2 diabetes mellitus (T2D), who have a 37% greater risk of developing CKD compared with males with diabetes (21). Women with CKD are also less likely to receive hemodialysis or renal transplant than men (22). Understanding the underlying causes of differences in CKD between men and women continues to be an important area of investigation.

Several medications are being evaluated for the treatment of CKD; however, women are often underrepresented in these clinical trials. This is concerning considering CKD’s greater prevalence in women (23). Additionally, there is uncertainty about cardiovascular outcomes for women in ongoing trials. SGLT2 inhibitors are reported to improve renal outcomes in CKD patients (24). While these trials suggest equal efficacy in diabetic men and women, most SGLT2 inhibitor trials enroll more men than women, and women appear to experience more frequent adverse events than men (25). It is noteworthy that SGLT2 inhibitor trials have not examined sex differences in nondiabetic populations (25, 26). Therefore, drawing conclusions about the efficacy of these drugs for treating disease in women should be questioned. Another compound under investigation for the treatment of CKD is the angiotensin receptor/neprilysin inhibitor (ARNI). ARNI treatment in CKD patients may confer additional benefit over ARB therapy. However, in studies of ARNI, 70% to more than 90% of the enrolled subjects were men, and data were not analyzed by sex (27, 28). Glucagon-like peptide 1 (GLP1) agonists and nonsteroidal mineralocorticoid receptor antagonists (MRAs) also show promise to slow CKD progression in T2D, but sex-specific responses to MRAs require further examination (29, 30). Ultimately, future clinical trials need to be designed and powered to fully assess safety and efficacy in both sexes.

### Key questions.

Despite efforts to improve the inclusion of women in clinical studies, women remain inadequately represented. BP target goals, specific antihypertensive regimens, and therapies have not been designed with sex-specific guidelines or recognition of key differences between men and women. Notably, many of the newer approaches to controlling BP have variable effects in men and women (SGLT2 inhibitors, ARNI, GLP1 agonists, nonsteroidal MRAs, and renal denervation). If studies are not powered to allow sex-specific conclusions, women will remain at a disadvantage. A more refined approach to treatment goals will be useful to inform the design of clinical trials testing novel antihypertensive therapies. Indeed, modeling approaches using NHANES data suggest that properly controlling pressure in hypertensive women has a greater impact on CVD mortality than it does in men, yet hypertension remains defined and treated as a one-size-fits-all disease (31).

## Hypertension clinical trials

Traditionally, representation of women in CVD trials is proportionally lower than the prevalence of the disease in women. In an analysis of 740 cardiovascular clinical trials completed between 2010 and 2017, only 38% of the participants were women (32). The participation to prevalence ratio (PPR) for the cardiovascular condition studied was assessed, with 1 representing trial representation to disease prevalence parity, and less than 0.8 defining underrepresentation. For hypertension trials, female PPR approached the definition of underrepresentation (ratio 0.82). The authors noted the importance of properly powering trials to draw meaningful conclusions about sex-specific interactions and outcomes. Given the common occurrence of hypertension in the population, this is a necessary step for obtaining valuable subgroup information from completed trials.

The Systolic Blood Pressure Intervention (SPRINT) trial included more than 9,000 hypertensive, nondiabetic individuals but did not stratify for sex at enrollment. The study compared the effectiveness of intensive BP control (SBP <120 mmHg) and standard treatment (SBP <140 mmHg). A significant benefit was reported in the intensive trial arm, with a 27% reduction in the primary outcome (fatal and nonfatal cardiovascular end points) (33). Foy et al. reanalyzed the SPRINT data to examine sex differences. At baseline, women took more total BP medications, and baseline SBP was 3 mmHg higher than in male participants (141 vs. 139 mmHg, respectively, *P* <0.001) (34). This aligns with observational data demonstrating higher routine BP readings among postmenopausal women (aged 60–69 years) and higher prevalence of hypertension than in men after the age of 60 (35). This subgroup analysis ultimately determined that the SBP control achieved was similar in men and women at the 3-year follow-up. Additionally, no significant interactions for the primary outcome, subcomponents, renal outcomes, or any of the recorded adverse events were reported (34). Although the authors suggested a possible heterogeneity of treatment effect for stroke (lack of benefit of intensive SBP lowering for women), the interaction terms were not statistically different in the overall effect. Wenger et al. (36), however, advised caution when interpreting SPRINT subgroup data, given the study’s failure to reach target recruitment rates for women, shorter follow-up intervals, and lower event rates among enrolled women.

Unlike SPRINT, the Action to Control Cardiovascular Risk in Diabetes (ACCORD) trial, which was focused on T2D patients, stratified for sex at enrollment (37). The BP substudy randomized 4,733 of the 10,251 participants to an intensive (SBP <120 mmHg) or standard (SBP <140 mmHg) BP treatment strategy (38). The confidence interval for the primary outcome (nonfatal myocardial infarction, nonfatal stroke, or death from cardiovascular causes) was greater than 1, indicating no benefit. While the heterogeneity analysis showed no difference between men and women with diabetes for the primary outcome, diabetes reportedly reverses the cardiovascular protection in young women: women with diabetes have poorer cardiovascular outcomes (39) and are more likely to be hypertensive than diabetic men (40, 41). This needs to be considered when analyzing the results of the ACCORD trial.

While the inclusion of women in recent hypertension trials is improving, device-based intervention trials continue to lag. An exciting development in treating resistant hypertension is the use of catheter-based (radiofrequency- or ultrasound-mediated) renal sympathetic innervation ablation, or renal nerve denervation (RND). In the long-term, 3-year follow up of the SYMPLICITY HTN-3 (RND in Patients with Uncontrolled Hypertension) trial, RND utilizing the Symplicity radiofrequency-based system (Medtronic) significantly reduced SBP compared with sham control treatment (42). While a heterogeneity analysis was not provided, the trial population included 39% women; yet few analyses have examined sex differences with this technology. Zweiker et al. reported that the likelihood of SBP reduction is 5-fold higher in women with RND compared with men (*P* < 0.006) (43). Whether the renal nerves play a more important role in women with resistant hypertension must be evaluated in larger clinical trials.

Guidelines for the treatment of hypertension in female-specific (i.e., pregnancy, postmenopause) or female-biased conditions (autoimmune diseases) are lacking. Studies such as the Chronic Hypertension and Pregnancy (CHAP) trial may begin to address questions surrounding female disease–specific BP control. The project randomized 2,419 pregnant women with treated or untreated chronic hypertension (present prior to 20 weeks of gestation) to two groups, one receiving therapy targeting a BP of less than 140 mmHg/90 mmHg, and the other receiving therapy with the same target only if “severe hypertension” (defined as SBP >160 mmHg, DBP >105 mmHg) developed”. Active treatment of chronic hypertension reduced the primary outcome risk by 18% (preeclampsia, medically indicated preterm birth before 35 weeks’ gestation, placental abruption, or fetal death) (44).

### Key questions.

Important questions remain regarding the effects of BP targets on key cardiovascular and renal outcomes, including stroke and CKD, that occur with higher prevalence in women. The effect of hypertension in older individuals also needs careful study, especially considering that women constitute a greater proportion (~55%) of the population older than 65 years as of the last US Census report. Studies of novel therapeutics, including device therapies, need to enroll women in proportion to disease prevalence to make conclusive decisions regarding safety and efficacy. The reasons for low enrollment of women may extend beyond experimental design. A recent meta-analysis concluded that older age and procedure invasiveness are likely barriers to women enrolling in trials (9). These barriers may be particularly relevant to hypertension trials given the high prevalence of hypertension in postmenopausal women and may partially explain the low participation of women in RND trials. Safety monitoring will also continue to be important in terms of fertility and pregnancy given the increasing number of women of childbearing years with hypertension (45).

## Genetic and chromosomal contributions to hypertension

### Human clinical evidence.

Hypertension risk is heritable (46), and an understanding of the genetic contribution to the development of hypertension and renal disease is evolving. Data from large hypertension study cohorts suggest that differences in hypertension prevalence cannot be explained exclusively by sex hormones (reviewed in refs. 47, 48).

The contribution of sex chromosomes to BP is imbalanced, with the coding capacity of the X chromosome far exceeding that of the Y chromosome. Although the Y chromosome largely encodes male gonadal development, Y chromosome genes are expressed in nongonadal tissues (49). Variations in the HindIII restriction site and expression of Y chromosome haplogroup I are associated with elevated BP in men (50). While limited data support a direct Y chromosome–mediated role for BP regulation, loss of the Y chromosome in blood cells is associated with reduced prevalence of hypertension, but a significant increase in cardiovascular events in a cohort of atherosclerotic men (51). In contrast, evidence suggests that X chromosomes influence hypertension susceptibility. Hypertension-related gene loci have been identified on the X chromosome (52, 53) which may explain the greater heritability of hypertension from the maternal side (46). X chromosome gene “dosing” occurs in both males and females (54, 55). Dysfunctional X inactivation in females, including upregulated long noncoding RNA X-inactive specific transcript (XIST) expression, promotes pulmonary arterial hypertension (56), increased cardiac events, endothelial dysfunction, and atherosclerosis (reviewed in ref. 57). Moreover, many genes “escape” X inactivation in females. Modeling approaches can help discern X inactivation effects using publicly available RNA sequencing data (58), thus positioning the field to better understand the contribution of this process to hypertension.

Salt sensitivity of BP may account for more than 50% of hypertension cases (59) and has an apparent genetic basis (60). Several large-scale clinical studies, including INTERSALT (61), Hypertensive Pathotype (HyperPath) (62), and GenSalt (63), report a higher prevalence of salt-sensitive hypertension in women than men. Salt-sensitive hypertension is also more prevalent in African American men and women compared with White Americans (64). One mechanism that may underly the greater salt sensitivity in women is enhanced aldosterone production in response to stimuli (e.g., angiotensin II [Ang II], low-salt diet, K^+^) than in men (62, 65, 66). Additionally, mutations in the *KCNJ5* gene, encoding an inward rectifying K^+^ channel, are more common in women than in men, and associate with inappropriate aldosterone production (67–69). Consistent with a greater role for aldosterone, MRA treatment lowers BP and improves all-cause mortality more effectively in women, supporting the hypothesis that there is a genetic basis for sex differences in aldosterone regulation and salt-sensitive hypertension.

### Preclinical studies.

In general, BP responses to hypertensive stimuli are more pronounced in male rodents than females (70), including in the Dahl salt-sensitive rat model, spontaneously hypertensive rats, Ang II–infused mice/rats, FVB/N mice, and 129Sv mice. Experimental models can be useful for exploring the mechanistic basis of hypertension. For example, BALB/c mice exhibit a female-specific elevation in BP in response to a high-salt diet, whereas neither female nor male C57BL/6 mice develop salt-sensitive hypertension (71). Strain differences can be exploited to understand the mechanisms of salt sensitivity. In this case, strain differences may arise from greater aldosterone production and aldosterone synthase expression in response to sodium restriction in female BALB/c compared with C57BL/6 mice (72–74). An acute sodium load is also reported to be more efficiently excreted in female mice (71) and rats (75) than in males, likely due to sex differences in the expression of electrolyte transporters across the nephron that are consistent with heightened sodium excretion in females. This demonstrates the importance of both sex and genetic background in rodent studies in the examination of BP control.

Unique preclinical models allow for the independent study of hormone and sex chromosome effects. The four-core genotype (FCG) mouse model (76) has been used for approximately 20 years, and the rat model is in development (77). The FCG model includes gonadal males and females with an XY or XX chromosome complement (78) allowing to assess the effects of sex chromosome complement, gonadal hormones, or their interaction. If chromosomes are found to mediate an effect, genetic crosses with the XY* model can then be employed to assess the contribution of X dose, X imprint or indirect effects of X inactivation, or the Y chromosome (79). This approach can be used to dissociate the complex interplay between hormones, chromosomes, and pathways central to the control of BP.

### Key questions.

Continued exploration of sex chromosome complement and hormonal control of BP is required. Experimental models such as the FCG, XY*, and genetic knockout models offer opportunities to dissect these mechanisms. In a world that is moving away from binary definitions of gender, gaining greater insight into how hormones and chromosomes work together to determine BP is critical (80, 81). Technological advances such as single-cell RNA sequencing can provide more mechanistic insight in preclinical and clinical studies, and tools such as CRISPR can rapidly generate novel models to allow further understanding of sex differences in hypertension.

## Immunological contributions to hypertension

Clinical data first linked hypertension with alterations in the immune system more than 60 years ago, when autoantibodies to vascular antigens were found in arterial samples of hypertensive cadaver specimens (82). Subsequent studies identified elevated levels of circulating IgG in hypertensive patients (83–85), and data from clinical and preclinical studies definitively link immune system dysfunction to hypertension.

### Human clinical evidence.

Immune cell population differences exist between men and women. Men have higher basal circulating immunoglobulin levels and greater numbers of B cells (85, 86) and CD4^+^ T cells (85). As a result, women typically respond better to vaccination and have less severe outcomes in infection than men. However, women are more likely to experience autoimmune disease or transplant rejection (87). In the context of hypertension, few studies have been designed to examine sex differences in immune parameters. Clinical studies found that cell populations from hypertensive subjects display a unique inflammatory phenotype compared with those from normotensive subjects, including monocytes (88), CD8^+^ T cells (89), and memory T cells (90), but none reported sex differences. Neutrophil-to-lymphocyte ratio (NLR) was measured in a large Taiwanese cohort to monitor its association with incident hypertension over 9 years. This study, stratified by sex, found that increased NLR associates with hypertension in older men (>60 years old), but not in women of any age (91). Another study assessing immune cell subsets in more than 4,000 women from Sister Study blood samples reported that those who developed hypertension had altered leukocyte populations prior to clinical diagnosis, specifically increased B cells and decreased naive CD4^+^ T cells (92), suggesting an immunological basis for hypertension in women. A large-scale Mendelian randomization study using UK Biobank population data found that increased circulating lymphocytes, monocytes, and neutrophils positively correlate with BP, but associations with sex, age, BMI, smoking, or alcohol intake were not detected (93). The authors acknowledged potential errors in the detection of confounding variables due to study design. Regardless, the potential to harness the immune system for improved BP control in men and women is intriguing.

### Hormonal contributions to immune system function.

Sex hormones influence immune cell differentiation, survival, and function (antibody production, cytokine secretion, phagocytic capacity) through estrogen and androgen receptors. Generally, endogenous estrogen enhances immune responses, while testosterone is immunosuppressive (94). The data on the interplay among sex hormones, immunomodulation, and BP in clinical research are primarily from studies involving hormone therapy. E2 binds to estrogen receptor α on T cells and inhibits proinflammatory cell expansion (95), resulting in protection from immune-mediated hypertension that is lost following menopause. Women receiving estrogen therapy have lower levels of soluble inflammatory markers including TNF-α, IL-6, and IL-8 and monocyte chemoattractant protein 1; but in general, estrogen therapy does not appear to significantly decrease BP in women (96–98). Data from the NHANES study show that men with testosterone deficiency have increased levels of circulating C-reactive protein (CRP), a marker of inflammation. Testosterone therapy reportedly lowers BP and decreases CRP (99) while reducing cardiovascular risk (99, 100). In contrast, other studies suggest that testosterone therapy in men may be detrimental to cardiovascular health (reviewed in ref. 101), although markers of immunomodulation were not widely reported. As hormone levels fluctuate over time and with age in men and women, understanding their impact on the immune system could be critically important to improving BP control.

### Preclinical studies.

Despite the extensive use of animal models, the impact of SABV on innate immune cells and their role in the development of hypertension remain poorly understood. Most studies examining the link between innate immune cells and hypertension include only male animals in the experimental design. Data suggest that male mice lacking macrophages have a blunted hypertensive response to Ang II infusion and DOCA-salt (deoxycorticosterone acetate–salt) hypertension (102, 103). While males accumulate more macrophages than females in several animal models of hypertension (104, 105), a role for macrophages in hypertension in females has not been examined. DCs are also pivotal for the development of hypertension in male mice. Salt and other hypertensive stimuli activate DCs, promoting oxidative stress, the formation of neoantigens, and T cell activation to promote hypertension (106, 107). Whether this mechanism is the same in female mice is unclear; however, plasmacytoid DCs from females produce more type I interferons, and estrogen drives DC differentiation from bone marrow precursors (108, 109), suggesting that DCs may have a similar role.

The association between the adaptive immune system and hypertension was definitively demonstrated in a study showing that male *Rag1^–/–^* mice, which lack mature B and T lymphocytes, have a blunted BP response to Ang II that is restored with adoptive transfer of T cells (110). Subsequent studies showed that Ang II increased BP when male, but not female, T cells were transferred into male *Rag1^–/–^* mice (111), demonstrating an important role for sex. The T cell subtype is also important in the development of hypertension, and sex differences in the T cell profile of males and females are notable. Males have more total T cells, CD4^+^ T cells, and renal Th17 cells, while females have more Tregs (112, 113). The increased percentage of renal Tregs in females may be compensatory to prevent further BP increases (114). Tregs also protect against DOCA-salt hypertension only in females (115), and restoration of Tregs in autoimmune-associated hypertension lowers BP in females (116). B cells also contribute to the development of Ang II hypertension in male mice. Mice with depleted or deficient B cells have an attenuated hypertensive response to Ang II (117). The role of B cells in hypertension has not been compared between the sexes; however, circulating B cells are altered with the development of hypertension in women (92), and B cells and immunoglobulin-secreting plasma cells have a pivotal role in the pathogenesis of autoimmune-mediated hypertension in females (118, 119).

### Key questions.

**Figure 2:**
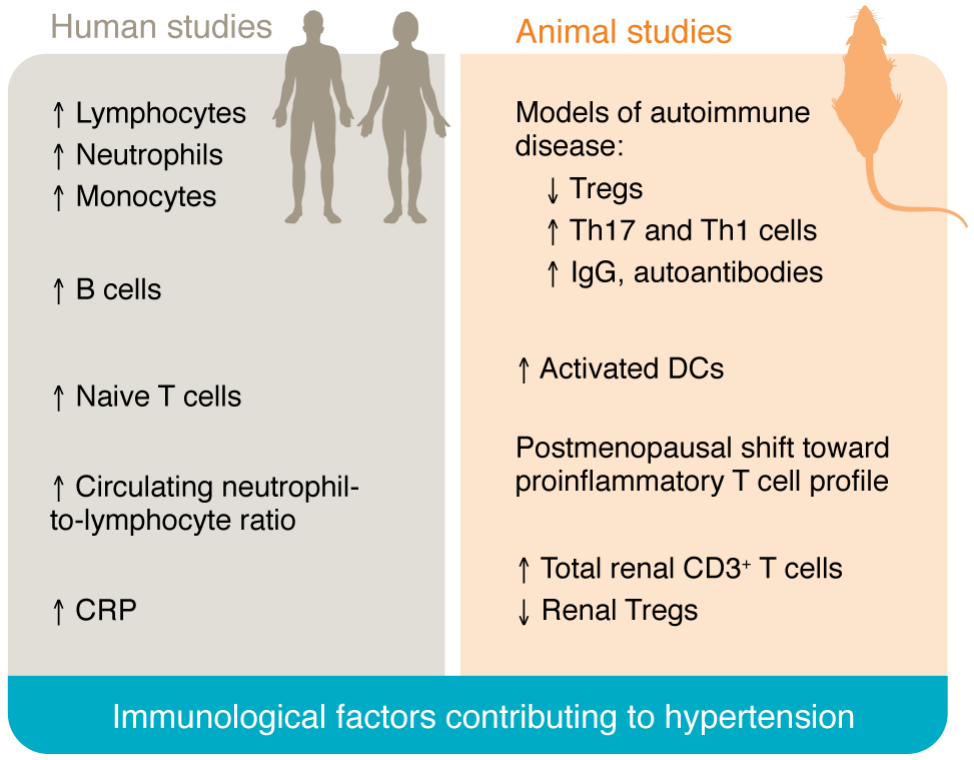
Summary of known immunological factors contributing to hypertension in men and women.

Preclinical models are instrumental for advancing our understanding of SABV in immune-mediated mechanisms of hypertension. However, research with large study populations is needed to understand the interplay among BP, sex hormones, and immunological changes, especially in populations not treated with hormonal interventions. Figure 2 summarizes major immunological changes known in human hypertension and preclinical models. Studies on the impact of antiinflammatory and immunomodulatory drugs on BP, including an analysis of the impact of SABV, are needed. In a secondary analysis of the Canakinumab Anti-inflammatory Thrombosis Outcomes Study (CANTOS) study, IL-1β inhibition did not reduce BP or incident hypertension; however, cardiovascular mortality, stroke, and MI were reduced in the hypertensive patients. This study did not stratify based on sex, and the population was largely (~75%) male (120). It is possible that antiinflammatory treatments work differently in males and females with respect to hypertension, particularly in pathologies that predominantly or exclusively affect women, such as autoimmunity or hypertension in pregnancy.

## Gut microbiota and hypertension

Studies in humans and experimental animal models implicate the gut microbiota in the development of hypertension, and the relationship among gut microbiota, sex differences, and hypertension has been extensively reviewed (121–124). However, few studies have specifically addressed SABV. Based on the potential of the gut microbiota to modulate BP, this is an active area of investigation (Table 2).

### Human clinical evidence.

Studies examining the human gut microbiota in men and women report differences in gut bacterial composition, with greater microbial diversity occurring in women. A multisite cross-sectional study was the first to report sex differences in the gut microbiome (125), with higher levels of the *Bacteriodes-Prevotella* phylogenetic group in men than women across the total study population. Others report differences in gut bacterial diversity between women and men, with many occurring in age- and hormone-dependent manners (126–129). Recently, a large-scale analysis of adults reported greater diversity in young adult women than age-matched men from the US, United Kingdom, and Columbia; this difference was not observed in middle-aged women (130).

It is increasingly evident that gut dysbiosis is common in both men and women with hypertension (131). Virwani et al. examined the association of sex and the gut microbiota with 24-hour ambulatory BP in 284 participants from Hong Kong, and found that gut microbiota dysregulation and beta diversity associated with hypertension in women but not men (132). In a study of nearly 2,000 individuals of African descent from five countries, the gut microbiota accurately predicted diabetes, glucose state, hypertension, obesity, and sex (133). The few human studies on hypertension and the gut microbiota clearly support an interwoven relationship between SABV and the gut microbiota.

### Hormonal contributions.

Sex steroids may contribute to the observed sexual dimorphism in the gut microbiota. Differences in gut bacterial groups and in predicted microbiota function, including the production of short-chain fatty acids, are observed in pre- and postmenopausal women (134). This supports the concept that hormone status regulates the gut microbiota in women. In women, bilateral ovariectomy and use of oral contraceptives associate with changes to specific bacteria (128). Further studies confirm that sex hormone levels correlate with gut microbial diversity and composition, uncovering both testosterone- and E2-responsive bacteria (135). While sex-specific gut microbiota function characterizes young adult men and premenopausal women, menopause and obesity appear to eliminate sex differences (136). Additional work is required to understand the complex mechanisms by which sex differences in the gut microbiota regulate BP.

### Preclinical studies.

Preclinical studies in rodents suggest that sex differences in gut microbiota composition do not occur until sexual maturation (137–139), supporting a key role for sex hormones. Sex hormone manipulation via gonadectomy induces microbial dysbiosis (140, 141) and eliminates sex differences in microbiota composition (138), with hormone administration reversing these effects (138, 140, 141). This is consistent with data from the literature on humans suggesting that sex-specific gut microbiome differences are reduced for postmenopausal women and age-matched men (136). Sex is a critical determinant of gut microbiota composition in hypertensive Dahl salt-sensitive rats and correlates with the degree of disease severity, independent of age, diet, or salt consumption (141). A comparison of germ-free versus conventionalized mice revealed that the gut microbiota modulates renal gene expression in a sex-specific manner (142). These differences likely affect BP regulation and potentially the efficacy of various therapeutics, as it was shown that fenbendazole treatment can sex-specifically change the gut microbiota in BPH/5 hypertensive/obese mice (143). Exciting work also explores the prospect of utilizing bioengineered, recombinant bacteria as treatment for hypertension: a recent study reported that *Lactobacillus* modified to express human ACE2 (hACE2) lowers BP in female, but not male, hypertensive *Ace2^–/–^* Dahl salt-sensitive rats (144).

### Key questions.

Research on the gut microbiota in health and disease is still in its infancy, and many unanswered questions remain. First, the impact of gonadal versus hormonal sex on microbiota composition in hypertension is unknown. The link between sex steroids, gonadal sex, and the gut microbiota, where estrogen-responsive bacteria have recently been identified, is being studied with the FCG model (145). However, FCG has yet to be used to understand the gut microbiota in hypertensive models. Second, while the microbiota–immune system interplay is evident in hypertension (141, 146–148), relevant sex differences are understudied. Finally, dietary components, including fat, salt, and protein, reportedly have sex-specific effects on hypertension and the immune system (141, 149, 150), and women are more susceptible to obesity and salt-induced increases in BP. Therefore, understanding the gut microbiota has the potential to provide novel insights into BP control in women.

## SABV in neural control of hypertension

The autonomic nervous system (ANS), through the integration of the parasympathetic and sympathetic branches, regulates essential physiological processes, including BP. ANS dysregulation leads to greater sympathetic outflow, blunted baroreflexes, and reduced cardiac vagal drive, resulting in hypertension (151, 152). Neural control of BP consists of a complex network that extends from the cerebral cortex to the spinal cord (153, 154). Thus, it is not surprising that there are multiple levels of sex-specific regulation in the neural control of BP. Sex differences are reported in adrenergic receptor function, subcellular distribution of neurotransmitter receptors, cellular responses to neurotransmitter release, and activation of baroreceptor circuits in response to external stimuli (153, 155). Consequently, ANS regulation of BP is fundamentally different between the sexes.

### Human clinical evidence.

Evidence of sex differences in human ANS regulation largely stems from measurements of muscle sympathetic nerve activity (MSNA) taken using microneurographic techniques. Sympathetic activity is higher in young men compared with age-matched women, with resting MSNA levels in men positively correlating with total peripheral resistance and inversely associating with cardiac output and α-adrenergic sensitivity (156–159). Relationships between resting MSNA and total peripheral resistance or MSNA are not detected in women, with arterial BP relying instead on β-adrenergic–mediated vasodilation to offset α-adrenergic vasoconstriction (158). Moreover, men appear to rely on MSNA bursts for BP maintenance, while women maintain BP throughout quiescent sympathetic periods (160), possibly due to a reduction in β-adrenergic sensitivity. These sex differences diminish in aged populations, as postmenopausal women are characterized by larger age-related increases in MSNA levels that essentially equal those of aged men (161). MSNA, BP, and total peripheral resistance are positively associated in both postmenopausal women and age-matched men (162), suggesting that women lose the compensatory effect of β-adrenergic vasodilation with age. These data highlight important differences in ANS regulation of BP that change over the lifespan.

### Hormonal contributions.

Sex hormones contribute to ANS regulation of BP, although in females they are more cyclically variable due to the estrous cycle. Sex hormone receptors have widespread, but sexually dimorphic, distributions in the brain (155). Notably, female risk for hypertension increases dramatically following menopause, when circulating E2 levels decline. In general, E2 binds estrogen receptors in ANS regulatory regions, leading to sympathoinhibitory effects. Estrogen receptor activation on both neurons and microglia in ANS regulatory brain regions contribute to a protective effect from hypertension in women (163). The effects of E2, however, are complex and context dependent, as E2 is necessary for the pathological cardiovascular effects observed in women exposed to psychosocial witness stress (164). While androgens are studied less and the findings are often mixed (165), they are often reported as exacerbating hypertension risk in males (166).

### Preclinical studies.

The use of rodent models has allowed for multiple genetic approaches to dissect circuits involved in hypertension, revealing important sex differences. Ang II administration to young male mice causes a slow progression toward hypertension not evident in age-matched female mice (167–169). This functional difference is likely due, in part, to sex differences in hypothalamic plasticity. Males and females exhibit opposing subcellular distribution patterns of the NMDA receptor GluN1 subunit, which alters localization in response to increased sympathetic outflow (170–173). Structural sex differences in the locus coeruleus, a brain region that is larger in females due to a greater number of norepinephrine-containing neurons (174), can also drive differences in ANS regulation of BP. Finally, there are sex differences in cortical regulatory centers such as the infralimbic (IL) region of the prefrontal cortex. Activation of IL projections in male and female rats reveals opposing sex-specific regulatory mechanisms, with stimulation lowering cardiac sympathetic drive and preventing chronic stress–induced cardiac remodeling in males (175–177). However, stimulation of the same projections in female rats facilitates sympathetic responses to stress, resulting in tachycardia and increased cardiac contractility after stress exposure (175).

### Key questions.

Our understanding of the interplay among stress, sex hormones, and neural circuits regulating BP is incomplete. The mechanisms underlying sex hormone mediation of ANS circuits remain unclear, particularly related to the effects of androgens and progesterone. Moreover, understanding of the regulation of the female endocrine system during and after chronic stress and across the lifespan is incomplete. Adolescence, pregnancy, postpartum, and menopause are understudied sexually dimorphic periods, particularly in neurophysiology, that can have lasting effects on cardiovascular health (13). Future studies that examine neural ANS regulatory circuits across critical periods are needed to understand female hypertension risk.

## Summary and conclusions

Concerted efforts continue to be made toward increasing SABV in experimental design, analysis, and reporting of clinical and basic science data. Evidence suggests that these efforts have improved inclusion of women in hypertension clinical trials since 2005 (178), although there remains work to be done, particularly with regard to device-based therapies (RND). Given that women were significantly underrepresented in clinical trials for the most widely used antihypertensive therapies, it is critically important to continue posing the question of the need for sex-specific guidelines in the treatment of hypertension.

**Figure 3:**
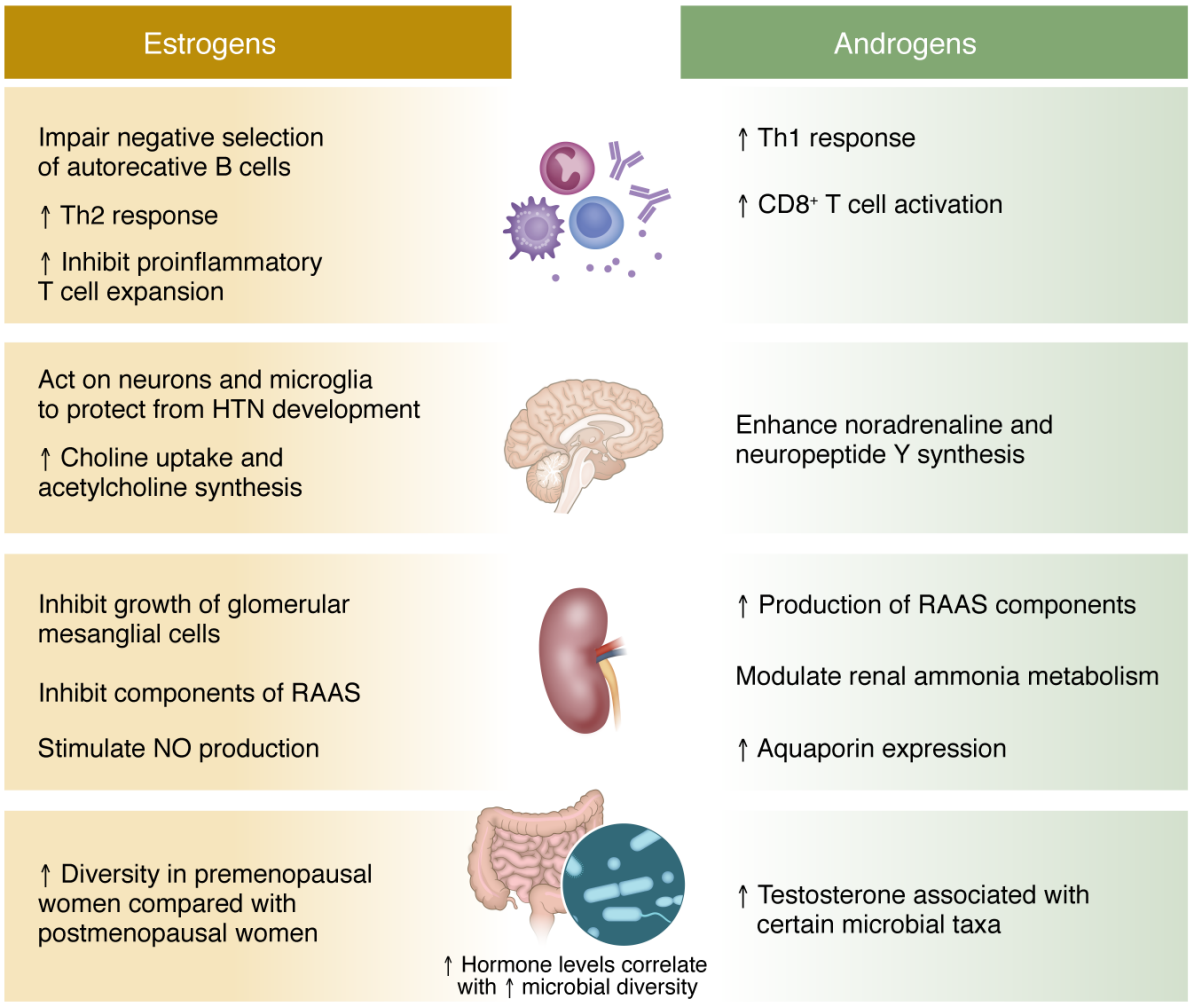
Summary of effects of estrogens and androgens on the immune system, central nervous system, kidneys, and gut microbiota that may affect blood pressure and the development of hypertension. HTN, hypertension; RAAS, renin angiotensin aldosterone system.

Both preclinical and clinical studies show a profound effect of sex steroids on organs and systems that control blood pressure (Figure 3), while progress is now being made toward improved understanding of sex chromosomes. Still, preclinical studies related to understanding BP control continue to lack the appropriate inclusion of SABV in experimental design, analysis, and reporting. Rectifying this is central to improving BP control in both men and women as we learn new information about the role of the immune system, the gut microbiota, and central mechanisms of BP control. Preclinical experimental models including the FCG mouse model and improved tools to understand the cellular and genetic basis of hypertension will be valuable for advancing the field. Through continued awareness campaigns and engagement/education at the level of funding agencies, individual investigators, and in the editorial peer review system, investigation of SABV in the field of hypertension research will ultimately lead to improved clinical outcomes. The collaboration of clinicians and preclinical researchers will further enhance the field, as clinical observations can inform questions in the laboratory, where mechanisms can be directly tested.

## Figures and Tables

**Table 2:**
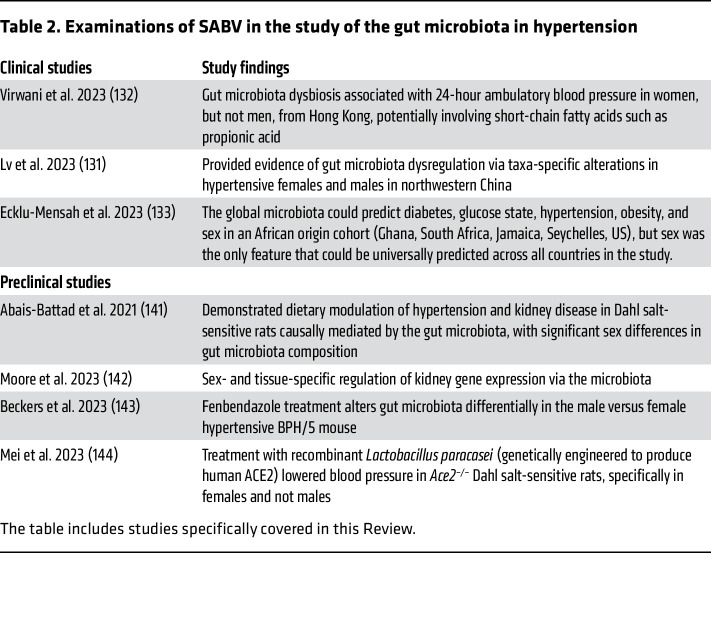
Examinations of SABV in the study of the gut microbiota in hypertension

**Table 1:**
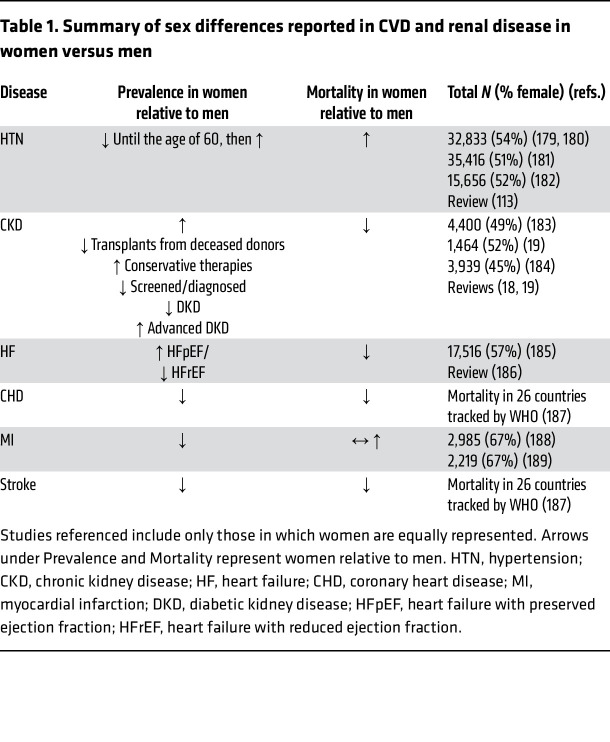
Summary of sex differences reported in CVD and renal disease in women versus men

## Version 1 09/03/2024

Electronic publication
